# Exploring the relationships between extreme body mass index and pain, somatic health, and psychiatric disorders in adolescents: a multinational analysis of general population data

**DOI:** 10.1186/s12889-026-30262-7

**Published:** 2026-11-04

**Authors:** Abdennour EL Mzadi, Btissame Zouini, Kourosh Bador, Maria Råstam, Nóra Kerekes

**Affiliations:** 1 Centre for Holistic Psychiatry Research (CHoPy), Mölndal, Sweden; 2 Department of Biology, Faculty of Sciences, Abdelmalek Essaadi University, Tetouan, Morocco; 3 Higher Institute of Nursing Professions and Health Techniques, Tetouan, Morocco; 4 Department of Clinical Sciences Lund, Lund University, Lund, Sweden; 5 Gillberg Neuropsychiatry Centre, Institute of Neuroscience and Physiology, University of Gothenburg, Gothenburg, Sweden; 6 Department of Health Sciences, University West, Trollhättan, 461 86 Sweden

**Keywords:** Adolescents, Body Mass Index, Cross-national study, Pain, Psychiatric disorders, Somatic health

## Abstract

**Background:**

Both high and low body mass index (BMI) in adolescence have been linked to physical and mental health problems, but these outcomes are rarely examined together. This study examined the associations between BMI categories and self-reported pain, somatic diseases, and psychiatric disorders among adolescents.

**Methods:**

A total of 5,132 adolescents from five countries (United States, Serbia, Sweden, Morocco, and Vietnam) participated in an online survey conducted between September 2020 and February 2021 as part of the Mental and Somatic Health Without Borders project. After excluding incomplete responses, data from 4,742 participants (mean age = 16.69 ± 1.02 years; 62.1% girls) were analysed. BMI categories were defined using WHO BMI-for-age z-scores, with normal BMI as the comparison group. Group differences were assessed using non-parametric and exact tests with multiple-comparison correction, followed by multivariable regression adjusted for age, gender, and country of residence.

**Results:**

Most participants had normal BMI (approximately 88%); 8.2% had overweight, 3.7% obesity, and 0.34% (*n* = 16) underweight. In adjusted analyses, adolescents with obesity had higher pain intensity and higher odds of reporting pain “all the time” than those in the comparison group. Overweight and obesity were associated with higher odds of asthma, while obesity was additionally associated with allergies and overweight with thyroid disease. Regarding psychiatric outcomes, overweight and obesity were associated with higher odds of anxiety, whereas obesity was additionally associated with higher odds of depression and attention-deficit/hyperactivity disorder. Findings involving underweight were less conclusive because of the small number of underweight adolescents and sparse events.

**Conclusion:**

Higher BMI categories were associated with distinct patterns of somatic, pain-related, and psychiatric outcomes among adolescents. Obesity showed the broadest pattern of associations, including greater pain burden and higher odds of several somatic and psychiatric conditions. These findings support considering BMI within a broader assessment of adolescent physical and mental health and highlight the value of integrated preventive strategies.

## Introduction

Body mass index (BMI), a widely used tool for assessing body weight, calculates an individual’s weight status by dividing their weight (in kilograms) by the square of their height (in meters). Although popularized in the 1950s by the Metropolitan Life Insurance Company, BMI was later critiqued by Keys et al.’s in 1972 for its limitations. These include its inability to distinguish between lean and fat mass and the failure to account for fat distribution [1]. Despite such limitations, BMI remains a widely used and convenient screening tool, originally developed for adults but now extended to children and adolescents [2, 3]. While for adolescents the same formula is used, BMI interpretation during this period requires age- and sex-specific reference standards due to rapid developmental changes. The World Health Organization (WHO) provides age- and sex-specific BMI-for-age growth references for children and adolescents aged 5–19 years, defining underweight (UW) as a BMI-for-age z-score < − 2 standard deviations (SD), normal weight as − 2 to + 1 SD, overweight (OW) as > + 1 to ≤ + 2 SD, and obesity (OB) as > + 2 SD [4].

Adolescence is a key developmental stage marked by rapid biological, psychological, and behavioral changes. During this period, body composition undergoes substantial variation, and susceptibility to both somatic and mental health conditions increases [5, 6]. Consequently, BMI deviations during adolescence may have lasting effects on health trajectories into adulthood [7].

From a global perspective, the prevalence of OW and OB in children and adolescents has surged dramatically, from 4% in 1975 to over 18% in 2016 [8]. Similar upward trends have been observed worldwide, underscoring the global burden of adolescent OW and OB [8–12]. In contrast, UW adolescents have received comparatively less attention, despite growing evidence of its continued relevance as a public health issue. Moderate to severe UW remains prevalent, particularly in low- and middle-income countries [8, 13]. Given the developmental importance of adolescence, extremes in BMI, whether low or high, can have enduring impacts on physical and mental health.

OW and OB are multifactorial conditions involving genetic predisposition [14–16] and environmental influences [17, 18]. They have been consistently associated with mental health issues, including depression [19–22], anxiety [20, 22, 23], low self-esteem [24, 25], and the disordered eating patterns such as bulimia nervosa and binge eating disorder [26, 27]. In addition, they are linked to somatic diseases, such as cardiovascular disease [28–30], type 2 diabetes [28, 31–33], and specific cancers [34, 35]. Conversely, adolescents with UW have also been shown to be at elevated risk for psychological difficulties. Studies by Tajik et al. and Zeiler et al. indicate heightened vulnerability to depression, negative body image [23, 36] and internalizing symptoms among UW youth [23, 37]. Physical consequences of UW in adolescents include reduced quality of life, poor physical fitness, amenorrhea, decreased bone mineral content, fatigue [23, 38–40], and altered pain perception [41]. Furthermore, UW has been associated with conditions such as epilepsy [42], celiac disease [43], rheumatologic diseases [44, 45], and cancers like leukemia [46], as well as restrictive eating disorders, including anorexia nervosa and avoidant/restrictive food intake disorder [26].

OB has also emerged as an independent risk factor for neuropathy, even in the absence of hyperglycemia [47]. Approximately one-third of adolescents with chronic pain also have OB [48, 49]. Recognized as chronic low-grade inflammatory condition, OB is linked to diabetes, cardiovascular disease, fibromyalgia, and chronic pain [50]. Fat tissue acts as neuroendocrine organ, releasing pro-inflammatory mediators that may contribute to pain [51].Gerdle et al. proposed that mechanical stress and neuroimmune interaction with fat pads contribute to chronic inflammation and sensory hypersensitivity [52]. A recent biomarker study suggests that OB and chronic pain may interact, especially in girls, through dysregulated inflammatory pathways [53].

Despite increasing recognition of the health risks associated with both ends of the BMI spectrum, the majority of existing studies have focused primary on OB and often examining physical or mental health outcomes in isolation. Few studies have simultaneously investigated UW, OW, and OB within a single adolescent cohort, particularly in a multinational context. Moreover, comparative data on pain, somatic conditions, and psychiatric disorders across these BMI categories remain limited.

The present study seeks to address this gap by utilizing a multinational sample of adolescents from countries with diverse cultural backgrounds, levels of economic development, and healthcare systems. Participants were recruited through the international “Mental and Somatic Health Without Borders” (MeSHe) project, which employs validated instruments to examine adolescent health across countries. This cross-national design enhances the generalizability of findings regarding BMI–health associations, overcoming the limitations of single-country studies. Accordingly, the study aimed to assess the prevalence of extreme BMI categories (UW, OW, OB) in a multinational adolescent sample and to compare self-reported pain, somatic diseases and complaints, and psychiatric disorders across BMI groups. By jointly examining both UW and OW conditions, the study provides an integrated understanding of how BMI extremes relate to adolescent health, moving beyond obesity-focused models.

### Aims

1. To estimate the prevalence of extreme BMI categories (UW, OW, and OB) in a multinational adolescent sample.
2. To compare self-reported pain (frequency and intensity), somatic diseases and complaints, and the diagnosed psychiatric disorders between adolescents within normal BMI and those with extreme BMI values.

## Method

### Study design

The present study is part of the international “Mental and Somatic Health without borders” (MeSHe) project (https://meshe.se/). This project employs a cross-sectional design, using a non-probabilistic sampling and structured self-report instruments for data collection. In each participating country, country-specific ethical approval was obtained in accordance with national requirements prior to administering the online survey to secondary upper high school students. Participants were recruited either through schools or via targeted social media campaigns (Facebook and Instagram) during the autumn semester 2020 (from September 2020 to February 2021). A comprehensive overview of the project methodology is available in Kerekes et al. [54], .

### Study population

Responses to the electronic survey (eMeSHe) were collected from 5,132 adolescents, aged 15 and 19. Data from 390 participants were excluded due to missing values on key variables required to calculate BMI-for-age z-scores (i.e., height, weight, or gender), or because they identified as non-binary. This latter group, (*n* = 58; 1.1% of the initial sample) was excluded because the World Health Organization (WHO) BMI-for-age reference standards are sex-specific and do not currently support z-score calculation for individuals outside the male/female binary. This group is described in more detail by Johansson et al. [55].

No imputation procedures were applied; all analyses were conducted using complete-case data. The final analytic sample consisted of 4,742 adolescents (mean [M] = 16.69; standard deviation [SD] = 1.02; median [Md] = 17) of whom 62.1% were girls (Table 1).

**Table 1:** Participant exclusions and gender distribution in the final study sample

Description	*N*	%
Adolescents who completed the survey	5132	100
Excluded total number of participants	390	7.6
– exclusion based on missing data on height, weight, gender	332	6.5
– exclusion based on gender reported “other than boy or girl”	58	1.1
Final study population included in analyses	4742	92.4
– Adolescent girls	2945	62.1
– Adolescent boys	1797	37.9

### Groups

BMI was calculated from self-reported weight and height provided by participants in the eMeSHe survey and expressed as kilograms per square meter (kg/m²). BMI-for-age z-scores were computed using WHO age- and sex-specific reference standards for individuals aged 5–19 years [4]. In this system, a z-score of 0 corresponds to the population mean, with cut-offs at ± 1 and ± 2 SD used to classify participants into BMI categories.

Based on WHO criteria, adolescents were categorized into four BMI groups as follows:

- ➢ Comparison group (CG): Adolescents with normal BMIs (*n* = 4163; 63% girls) defined as BMI-for-age z-scores between − 2SD and + 1 SD.
- ➢ Underweight (UW): Adolescents classified as having thinness or severe thinness (*n* = 16; 62.5% boys), defined as BMI-for-age z-scores < − 2 SD.
- ➢ Overweight (OW): Adolescents with overweight (*n* = 387; 54.5% girls), defined as BMI-for-age z-scores > + 1 SD and ≤ + 2 SD.
- ➢ Obesity (OB): Adolescents with obesity (*n* = 176; 60.8% girls), defined as BMI-for-age z-scores > + 2 SD.

### Instruments

The eMeSHe survey includes eight questionnaires (see www.meshe.se) along with background items that collect information on each participant’s age, gender identity, weight, height, country of residence, diagnosed psychiatric disorders, and perceived pain. These background variables were utilized for the present study. Across all participating countries the eMeSHe survey was administered using the same standardized questionnaires to ensure cross-country comparability. Country-specific language versions of the questionnaires were administered in each participating country to ensure linguistic accessibility for all participants. Translation into each national language was carried out by national coordinators with relevant clinical and/or academic backgrounds, ensuring accurate rendering of diagnostic and medical terminology. Given that the background variables analyzed in this study are numeric, binary, or based on standard medical terminology rather than attitudinal or culturally variable constructs, formal cross-cultural adaptation procedures (e.g., cognitive debriefing) were not considered necessary beyond ensuring translation accuracy. Additionally, data from one of the eight questionnaires, the *MeSHe health survey*, were analyzed. This instrument contains items assessing the presence of the following medically diagnosed somatic diseases: asthma, other allergies, cancer/leukemia or other tumor diagnosis, celiac disease, diabetes, epilepsy, migraine, rheumatological diseases, skin disease, thyroid diseases, tuberculosis, as well as prolonged gastrointestinal complaints, defined as constipation or diarrhea lasting longer than 14 days. The *MeSHe health survey* has previously been used and validated in adolescent populations [56, 57], demonstrating high test-retest reliability [56].

### Statistical methods

All statistical analyses were performed using IBM SPSS Statistics (version 28). Categorical variables were summarized using frequencies and percentages. The distribution of BMI values was assessed using the Kolmogorov–Smirnov test, which indicated a significant deviation from normality (*p* < 0.001); therefore, non-parametric tests were applied. Differences in the prevalence of somatic conditions, psychiatric diagnoses, and pain-frequency categories between each extreme BMI category (UW, OW, and OB) and the comparison group (CG) were examined using two-sided Fisher’s exact tests. The Benjamini–Hochberg false discovery rate procedure was applied to account for multiple comparisons. Effect sizes were assessed using Cramer’s V and interpreted as follows: values close to 0 indicated a negligible effect, 0.05–0.10 a weak effect, 0.10–0.15 a moderate effect, 0.15–0.25 a strong effect, and > 0.25 a very strong effect [58].

Adjusted associations between BMI category and binary somatic and psychiatric outcomes were examined using multivariable logistic regression, whereas pain frequency was analyzed using multinomial logistic regression because the proportional-odds assumption for ordinal regression was not met. The CG served as the reference BMI category, with “never” as the reference outcome for pain frequency. Adjusted odds ratios (aORs) with 95% confidence intervals (CIs) were reported. When zero events in the UW group resulted in complete separation, models were re-estimated without this category; when sparse data or separation otherwise prevented reliable estimation, adjusted estimates were not reported. All multivariable models were adjusted for age, gender, and country of residence.

Pain intensity was compared across BMI categories using the Kruskal–Wallis test, followed by Bonferroni-adjusted pairwise comparisons, with epsilon squared (ε²) used to quantify the overall effect size. Epsilon squared was calculated from the H-statistic as ε² = (H − k + 1)/(n − k), where *H* is the test statistic, *k* is the number of groups, and *n* is the total sample size. A generalized linear model with a normal distribution and identity link was subsequently used to estimate adjusted mean pain scores and 95% CIs, with pairwise comparisons against the CG corrected using the Bonferroni method. Statistical significance was set at *p* < 0.05 after correction for multiple comparisons where applicable.

## Results

### The prevalence of BMI categories in the multinational adolescent sample

Of the adolescents in the final study population, approximately 88% were classified in the comparison group (CG) based on normal BMI-for-age z-scores. The remaining participants were categorized as overweight (OW; 8%), obese (OB; 3.6%), or underweight (UW; approximately 0.3%) according to WHO BMI-for-age z-score cut-offs ( < − 2 SD for underweight, > +1 SD to ≤ + 2 SD for overweight, and > + 2 SD for obesity) (Table 2).

**Table 2:** Distribution of BMI categories in the multinational adolescent study population, overall and by gender

	All participants (*n* = 4742) %	Adolescent Boys (*n* = 1797) %	Adolescent Girls (*n* = 2945) %	χ^2^ Test		
				χ^2^	*p*	V
UW	0.34	0.56	0.20	4.13	0.04	0.03
CG	87.79	85.81	89.00	10.59	0.001	0.05
OW	8.16	9.79	7.16	10.3	0.001	0.05
OB	3.71	3.84	3.63	0.13	0.72	-

*BMI* body mass index, *CG* comparison group, *OB* obesity, *OW* overweight, *UW* underweight, *V* Cramer’s *V*. Each BMI category was compared between boys and girls with a separate 2 × 2 χ² test

Chi-squared tests revealed significant gender differences in the distribution of extreme BMI categories. Adolescent boys were more likely to be classified as UW (0.56%) or OW (9.79%) compared with adolescent girls (*p* = 0.04 and *p* = 0.001, respectively), associations were characterized by weak effect sizes. Correspondingly, adolescent girls were more likely to be classified within CG (*p* < 0.001), also with weak effect size (Table 2).

### The prevalence of somatic diseases and complaints by BMI categories

In the unadjusted analyses, several differences in somatic conditions were observed between the BMI groups and the CG (Table 3). After correction for multiple comparisons, overweight adolescents more frequently reported diarrhea (8.18% vs. 4.04%, *p*adj = 0.013), asthma (13.37% vs. 8.51%, *p*adj = 0.023), and thyroid disease (3.19% vs. 1.11%, *p*adj = 0.023) than adolescents in the comparison group. Among adolescents with obesity, constipation (13.92% vs. 7.19%, *p*adj = 0.028), asthma (18.79% vs. 8.51%, *p*adj < 0.010), and allergies (37.01% vs. 23.37%, *p*adj < 0.010) were more frequently reported than in the CG. The effect sizes for these differences were small (Cramer’s V = 0.048–0.070).

**Table 3:** Prevalence of self-reported somatic diseases and complaints across BMI categories

Somatic disease	CG (%)	UW (%)	*p*	padj	V	OW (%)	*p*	padj	V	OB (%)	*p*	padj	V
Allergies	23.37	13.33	0.543	0.843	—	27.68	0.078	0.217	—	37.01	< 0.001	< 0.010	0.062
Asthma	8.51	6.25	1.000	1.000	—	13.37	0.003	0.023	0.048	18.79	< 0.001	< 0.010	0.070
Cancer	0.57	12.50	0.004	0.026	0.096	0.27	0.717	0.902	—	0.58	1.000	1.000	—
Constipation	7.19	0.00	0.620	0.864	—	10.03	0.065	0.211	—	13.92	0.005	0.028	0.050
Diabetes	0.59	0.00	1.000	1.000	—	1.07	0.292	0.599	—	1.20	0.274	0.594	—
Diarrhea	4.04	6.25	0.485	0.788	—	8.18	0.001	0.013	0.055	7.79	0.036	0.128	0.036
Epilepsy	0.67	12.50	0.006	0.029	0.088	1.07	0.330	0.644	—	1.79	0.117	0.304	—
Gluten intolerance	1.15	6.25	0.171	0.417	—	1.90	0.208	0.477	—	1.23	0.711	0.902	—
Migraine	8.93	12.50	0.649	0.873	—	9.80	0.562	0.843	—	10.76	0.397	0.737	—
Rheumatologic disease	1.11	0.00	1.000	1.000	—	2.20	0.076	0.217	—	1.88	0.428	0.744	—
Skin disease	11.42	31.25	0.029	0.125	0.040	10.96	0.863	1.000	—	6.10	0.032	0.125	—
Thyroid disease	1.11	0.00	1.000	1.000	—	3.19	0.003	0.023	0.052	0.60	1.000	1.000	—
Tuberculosis	0.32	0.00	1.000	1.000	—	0.00	0.619	0.864	—	0.59	0.439	0.744	—

*CG* comparison group, *UW* underweight, *OW* overweight, *OB* obesity, *V* Cramer’s *V*; *p*  two-sided Fisher’s exact test p-value, *p*adj Benjamini–Hochberg false discovery rate-adjusted p-value

Cancer/tumour diagnosis and epilepsy were also more frequently reported in the UW group (12.50% vs. 0.57%, *p*adj = 0.026; and 12.50% vs. 0.67%, *p*adj = 0.029, respectively). However, both findings were based on only two cases among 16 UW adolescents, compared with 23/4021 and 27/4017 adolescents in the comparison group, respectively. Given these sparse data, these findings should be interpreted cautiously and are presented primarily as descriptive observations rather than robust evidence of an association. No significant differences after correction for multiple comparisons were observed for the remaining somatic conditions.

**Fig. 1:**
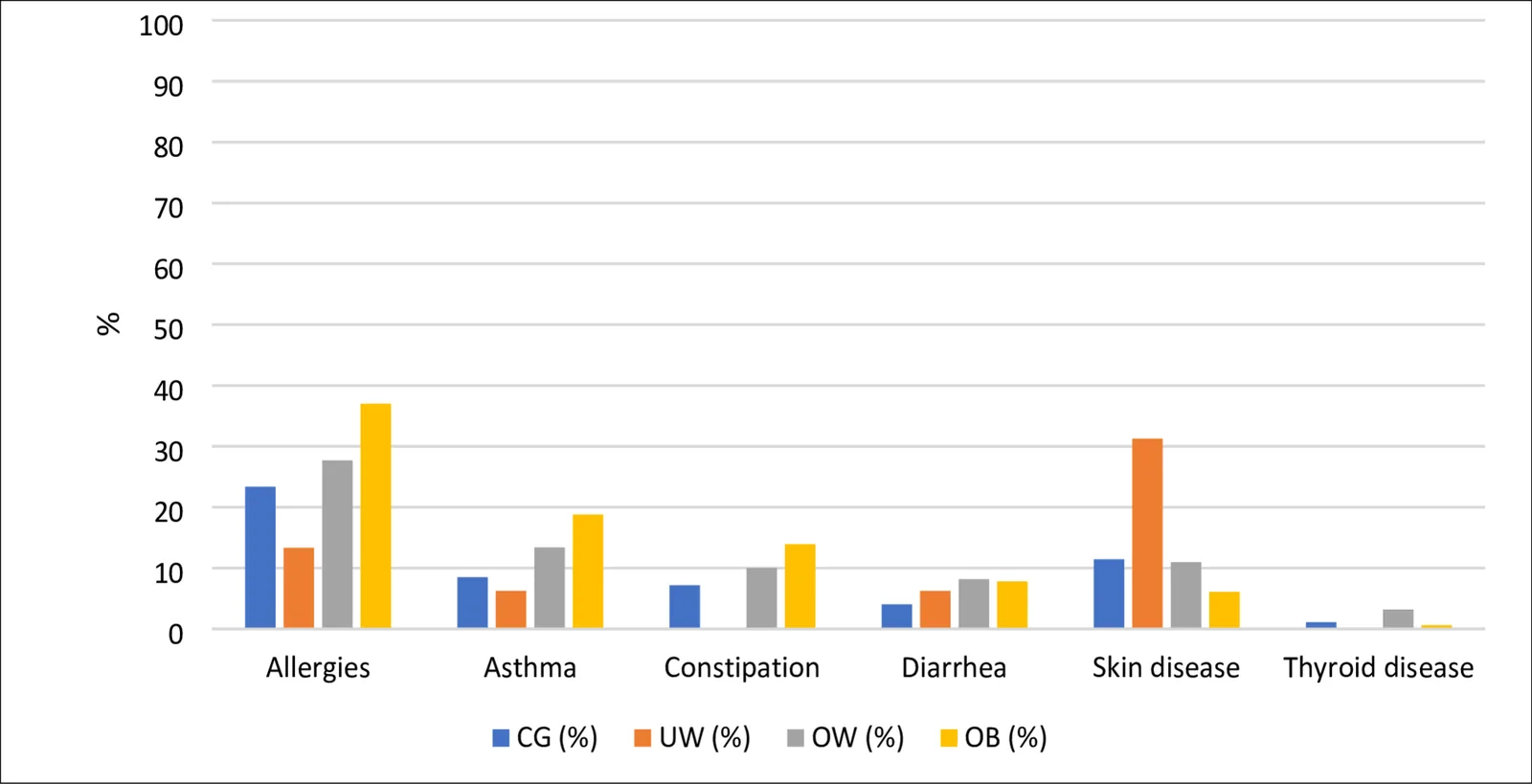
Prevalence of selected somatic diseases and complaints across BMI categories in adolescents

Figure 1 presents the percentage of adolescents reporting selected somatic diseases and complaints in the CG, and in the UW, OW, and OB groups. Percentages were calculated from the available observations for each outcome, so denominators may differ between conditions.

After adjustment for age, gender, and country of residence, the pattern of associations was more limited.BMI category remained significantly associated with four outcomes: asthma (overall *p* = 0.02), allergies (*p* = 0.04), skin disease (*p* = 0.04), and thyroid disease (*p* = 0.01) (Table 4).

**Table 4:** Adjusted associations between BMI categories and somatic health outcomes

Somatic outcome	UW vs. CG aOR (95% CI)	*P*	OW vs. CG aOR (95% CI)	*p*	OB vs. CG aOR (95% CI)	*p*	Overall BMI *p*
Asthma	0.62 (0.08–4.87)	0.65	1.40 (1.01–1.94)	0.04	1.74 (1.13–2.66)	0.01	0.02
Allergies	0.42 (0.09–1.9)	0.26	1.10 (0.86–1.41)	0.45	1.56 (1.10–2.22)	0.01	0.04
Skin disease	3.66(1.23–10.91)	0.02	1.10 (0.78–1.57)	0.58	0.61 (0.32–1.19)	0.15	0.04
Thyroid disease	NE	—	2.87 (1.47–5.58)	0.002	0.46 (0.06–3.51)	0.45	0.01
Diarrhea	1.16 (0.15–9.29)	0.89	1.69 (1.10–2.62)	0.02	1.40 (0.74–2.65)	0.30	0.10
Constipation	NE	—	1.30 (0.89–1.92)	0.18	1.57 (0.96–2.58)	0.075	0.21
Diabetes	NE	—	1.53 (0.52–4.49)	0.44	2.38 (0.55–10.31)	0.25	0.62
Migraine	1.06 (0.23–4.75)	0.95	1.19 (0.82–1.73)	0.37	1.14 (0.66–1.97)	0.64	0.81
Rheumatologic disease	NE	—	2.05 (0.95–4.44)	0.07	1.79 (0.52–6.16)	0.36	0.29
Gluten intolerance	5.64 (0.67–47.65)	0.11	1.48 (0.66–3.32)	0.35	0.82 (0.19–3.56)	0.80	0.33

*aOR* adjusted odds ratio, *CI* confidence interval, *CG* comparison group, *UW* underweight, *OW* overweight, OB obesity, *NE* not estimable. Models were adjusted for age, gender, and country of residence. *p*-values are from the corresponding multivariable logistic regression models

Adolescents with OW and OB had higher odds of reporting asthma than those in the CG (aOR = 1.40, 95% CI: 1.01–1.94 and aOR = 1.74, 95% CI: 1.13–2.66, respectively). Similarly, adolescents with OB had higher odds of reporting allergies (aOR = 1.56, 95% CI: 1.10–2.22). The estimates for the UW group were substantially less precise, reflecting its small sample size and the very small number of events.

For skin disease, higher odds were observed in the UW group (aOR = 3.66, 95% CI: 1.23–10.91), although the estimate was imprecise because of the small number of underweight participants. For thyroid disease, higher odds were observed among adolescents with OW (aOR = 2.87, 95% CI: 1.47–5.58), whereas no clear association was observed for OB. An adjusted estimate could not be obtained for UW because no thyroid disease cases were reported in this group.

The higher prevalence of diarrhea observed among adolescents with OW in the unadjusted analysis persisted after adjustment (aOR = 1.69, 95% CI: 1.10–2.62, *p* = 0.02). However, the overall adjusted association between BMI category and diarrhea was not statistically significant (*p* = 0.10). No overall adjusted associations were observed for constipation, diabetes, migraine, rheumatologic disease, or gluten intolerance.

Cancer/tumour diagnosis, epilepsy, and tuberculosis were not included in the adjusted models because of sparse events and were therefore retained as descriptive findings.

### Prevalence and intensity of perceived pain by BMI categories

A significant association was found between pain frequency and BMI categories (χ² [12] = 77.64; *p* < 0.001), although the effect size was weak (Cramer’s V = 0.074). Participants were asked whether they had experienced pain in the previous 12 months, selecting from *never*,* rarely*,* sometimes*,* often*, or *all the time*. Compared to the CG (31.95%), adolescents in the OW (21.67%) and OB (19.32%) groups were significantly less likely to report “rarely” experiencing pain (BH-adjusted *p* < 0.005 for both, with small effect sizes: V = 0.062 and V = 0.054, respectively) (Table 5).

**Table 5:** Frequency of perceived pain in adolescents, by BMI categories

Pain frequency	CG (%)	UW (%)	*p*	padj	V	OW (%)	*p*	padj	V	OB (%)	*p*	padj	V
Never	20.02	0.00	0.053	0.131	—	21.15	0.594	0.636	—	15.34	0.147	0.221	—
Rarely	31.95	20.00	0.414	0.478	—	21.67	< 0.001	< 0.005	0.062	19.32	< 0.001	< 0.005	0.054
Sometimes	36.08	60.00	0.063	0.131	—	40.21	0.120	0.200	—	44.32	0.031	0.108	—
Often	10.22	13.33	0.661	0.661	—	13.84	0.036	0.108	—	12.50	0.312	0.390	—
All the time	1.73	6.67	0.232	0.316	—	3.13	0.070	0.131	—	8.52	< 0.001	< 0.005	0.096

*CG* comparison group, *UW* underweight, *OW* overweight, *OB* obesity V Cramer’s V; *p*  two-sided Fisher’s exact test *p*-value; *p*adj = Benjamini–Hochberg false discovery rate-adjusted *p*-value

Importantly, adolescents in the OB group were significantly more likely to report experiencing pain “all the time” compared to those in the CG (8.52% vs. 1.73%; BH-adjusted *p* < 0.005, V = 0.096) (Table 5).

After adjustment for age, gender, and country of residence, BMI category remained significantly associated with pain frequency (likelihood-ratio χ² [12] = 31.253, *p* = 0.002). Compared with the CG, adolescents with OB had higher odds of reporting pain “all the time” rather than “never” (aOR = 3.58, 95% CI: 1.72–7.46, *p* = 0.001) (Table 6). No significant differences were observed between the OW and CG groups for any pain-frequency category, or between the OB and CG groups for “rarely,” “sometimes,” or “often” experiencing pain. Estimates for the UW group were not considered reliable because of quasi-complete separation resulting from the very small group size and zero cells.

**Table 6:** Adjusted associations between BMI category and pain frequency

Pain frequency	UW vs. CG aOR (95% CI)	*p*	OW vs. CG aOR (95% CI)	*p*	OB vs. CG aOR (95% CI)	*p*
Never	Reference outcome	—	Reference outcome	—	Reference outcome	—
Rarely	NE	—	0.77 (0.56–1.07)	0.117	1.04 (0.61–1.76)	0.888
Sometimes	NE	—	1.06 (0.79–1.42)	0.688	1.51 (0.95–2.41)	0.083
Often	NE	—	1.18 (0.81–1.73)	0.393	1.34 (0.73–2.47)	0.341
All the time	NE	—	1.48 (0.76–2.90)	0.249	3.58 (1.72–7.46)	0.001

*aOR* adjusted odds ratio, *CI* confidence interval, *CG* comparison group, *UW* underweight, *OW* overweight, *OB* obesity, *NE* not reliably estimable

**Fig. 2:**
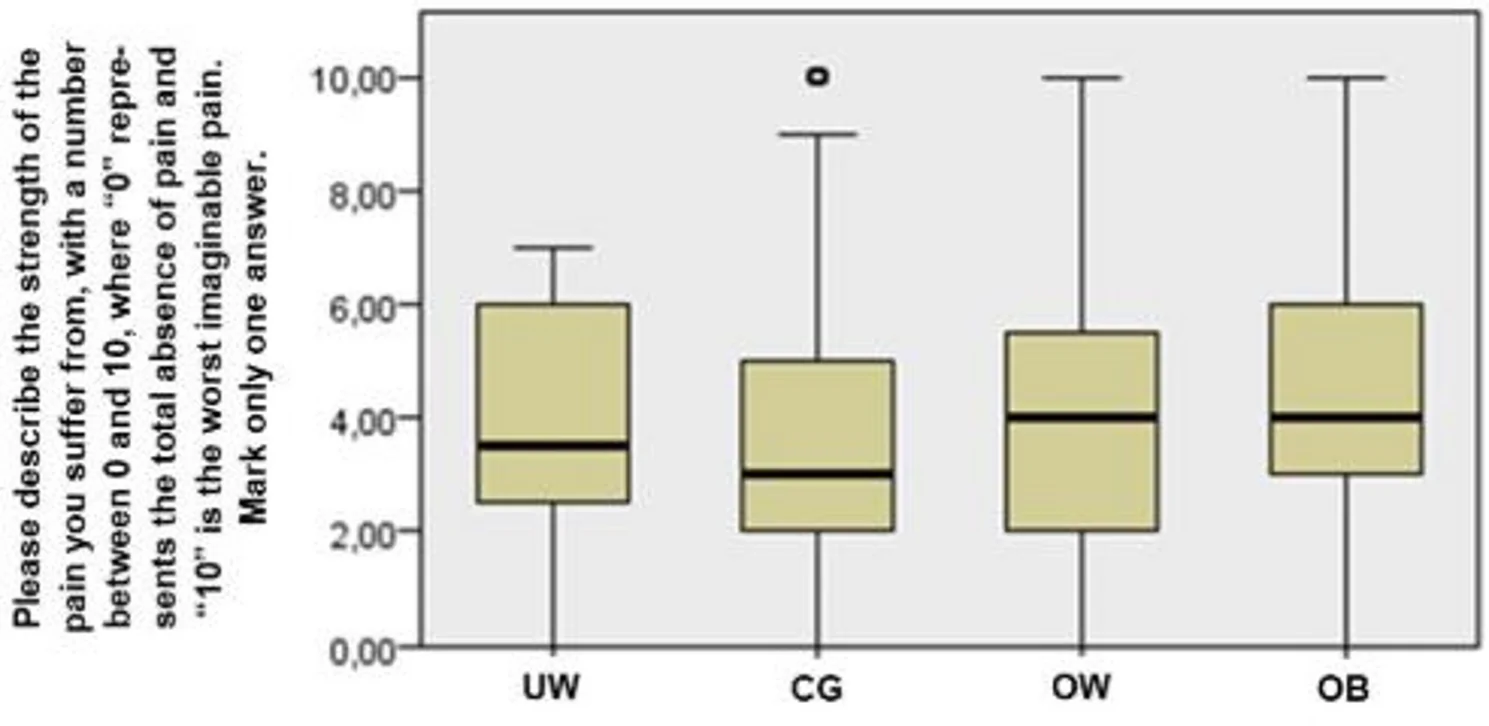
Self-reported pain intensity across BMI categories in adolescents. Note: CG=comparison group; OB=obesity; OW=overweight; UW=underweight

Self-reported pain intensity differed significantly across BMI categories in the unadjusted analysis (Kruskal–Wallis *H* [3] = 8.101, *p* = 0.044; Fig. 2), although the effect size negligible (ε² = 0.001) (Table 7).

After Bonferroni correction, only the OB group differed significantly from the CG, with higher pain intensity in the OB group (Bonferroni-adjusted *p* = 0.039). The difference between the OB and OW groups was nominally significant before correction but not after. No other pairwise comparisons were statistically significant after correction (Table 7).

**Table 7:** Unadjusted differences in pain intensity across BMI categories

	BMI categories				Pairwise comparison	Test statistic	Unadjusted *p*	Bonferroni-adjusted *p*	ε²
	CG	UW	OW	OB					
Mean rank of pain intensity	2096.09	2331.72	2132.92	2363.53	OB > CG	−267.44	0.006	0.039	
					OB > OW	−230.61	0.047	0.283	
					UW > CG	235.63	0.437	1.000	
					UW > OW	198.80	0.520	1.000	
					OW > CG	−36.83	0.589	1.000	
					OB > UW	−31.81	0.920	1.000	
					Overall Kruskal–Wallis	H = 8.101; df = 3	0.044	—	0.001

*CG* comparison group, *UW* underweight, *OW* overweight, *OB* obesity, *ε²* epsilon squared. Pairwise comparisons are presented with unadjusted and Bonferroni-adjusted *p*-values

After adjustment for age, gender, and country of residence, the overall association between BMI category and pain intensity remained statistically significant (Wald χ² [3] = 7.985, *p* = 0.046) (Table 8). Adolescents with OB had a higher adjusted mean pain score than those in the CG (4.06 vs. 3.55; Bonferroni-adjusted *p* = 0.028). No significant differences were observed between the UW and CG groups or between the OW and CG groups after Bonferroni correction. The estimate for the UW group was less precise, as indicated by its wider confidence interval, reflecting the small number of participants in this group.

**Table 8:** Adjusted pain intensity across BMI categories

BMI category	Adjusted mean pain score	95% CI	Uncorrected pairwise *p* vs. CG	Bonferroni-adjusted *p* vs. CG	
CG/Normal	3.55	3.45–3.65	Reference		Reference
UW	4.26	3.11–5.41	0.231		0.694
OW	3.60	3.35–3.85	0.736		1.000
OB	4.06	3.69–4.43	0.009		0.028
Overall BMI effect	Wald χ²(3) = 7.985, *p* = 0.046	—	—		—

Adjusted means were estimated controlling for age, gender, and country of residence. *CI* confidence interval, *CG* comparison group, *UW* underweight, *OW* overweight, *OB* obesity. Pairwise comparisons with the CG were Bonferroni-adjusted

### Prevalence of diagnosed psychiatric disorders by BMI categories

The prevalence of self-reported diagnosed psychiatric disorders varied across BMI categories (Table 9). No significant differences were observed between the UW and CG groups. After correction for multiple comparisons, anxiety (9.30% vs. 4.37%, padj < 0.010) and depression (8.53% vs. 5.02%, padj = 0.036) were more frequently reported in the OW group than in the CG, although the effect sizes were negligible (Cramer’s V = 0.064 and 0.044, respectively).

**Table 9:** Self-reported psychiatric diagnoses in adolescents across BMI categories

Psychiatric diagnosis	CG (%)	UW (%)	*p*	padj	V	OW (%)	*p*	padj	V	OB (%)	*p*	padj	V
ADD/ADHD	2.59	12.50	0.065	0.195	0.038	3.36	0.406	0.624	0.013	11.93	< 0.001	< 0.010	0.108
Anxiety	4.37	0.00	1.000	1.000	0.013	9.30	< 0.001	< 0.010	0.064	16.48	< 0.001	< 0.010	0.111
ASD	0.77	6.25	0.119	0.298	0.038	1.29	0.239	0.478	0.016	2.84	0.016	0.096	0.044
Bipolar disorder	0.22	0.00	1.000	1.000	0.003	0.26	0.589	0.736	0.003	1.14	0.071	0.194	0.036
Depression	5.02	0.00	1.000	1.000	0.014	8.53	0.006	0.036	0.044	18.75	< 0.001	< 0.010	0.118
Eating disorder	2.07	0.00	1.000	1.000	0.009	0.78	0.084	0.210	0.026	3.41	0.274	0.514	0.018
NDD-UNS	0.14	0.00	1.000	1.000	0.002	0.00	1.000	1.000	0.011	0.00	1.000	1.000	0.008
OCD	0.34	0.00	1.000	1.000	0.004	0.26	1.000	1.000	0.004	0.00	1.000	1.000	0.012
PTSD	0.65	0.00	1.000	1.000	0.005	1.81	0.022	0.110	0.038	0.57	1.000	1.000	0.002
Other (PD, gender dysphoria)	0.34	0.00	1.000	1.000	0.004	0.00	0.625	0.750	0.017	0.57	0.463	0.662	0.008

*ADD/ADHD* attention deficit disorder/attention-deficit/hyperactivity disorder, *ASD* autism spectrum disorder, *CG* comparison group, NDD-UNS neurodevelopmental disorder, unspecified, *OB* obesity, *OCD* obsessive-compulsive disorder, *OW* overweight, *PD* personality disorders, *PTSD* post-traumatic stress disorder, *UW* underweight, *V* Cramer’s *V,p*: two-sided Fisher’s exact test; *p*adj: *p*-value adjusted for multiple comparisons using the Benjamini–Hochberg false discovery rate method

Among adolescents with OB, ADD/ADHD (11.93% vs. 2.59%), anxiety (16.48% vs. 4.37%), and depression (18.75% vs. 5.02%) were significantly more frequently reported than in the CG, with small effect sizes (Cramer’s V = 0.108–0.118). Although ASD in the OB group and PTSD in the OW group were more frequently reported than in the CG, these differences did not remain significant after correction (Table 9).

After adjustment for age, gender, and country of residence, BMI category remained associated with ADD/ADHD, anxiety, and depression (Table 10). Adolescents with OB had higher odds of reporting ADD/ADHD than those in the CG (aOR = 2.19, 95% CI: 1.27–3.78), whereas no association was observed for OW. The estimate for UW was imprecise, with a wide confidence interval reflecting the small size of this group.

**Table 10:** Adjusted associations between BMI categories and self-reported psychiatric diagnoses

Psychiatric diagnosis	UW vs. CG aOR (95% CI)	*p*	OW vs. CG aOR (95% CI)	*p*	OB vs. CG aOR (95% CI)	*p*	Overall BMI *p*
ADD/ADHD	1.56 (0.31–7.89)	0.592	0.93 (0.51–1.70)	0.813	2.19 (1.27–3.78)	0.005	0.038
Anxiety	NE	—	1.83 (1.24–2.71)	0.003	2.10 (1.30–3.39)	0.002	< 0.001
ASD	NE	—	NE	—	NE	—	NE
Bipolar disorder	NE	—	NE	—	NE	—	NE
Depression	NE	—	1.45 (0.98–2.17)	0.066	2.31 (1.47–3.63)	< 0.001	0.001
Eating disorder	NE	—	0.30 (0.09–0.95)	0.041	0.94 (0.38–2.33)	0.896	0.124
NDD-UNS	NE	—	NE	—	NE	—	NE
OCD	NE	—	NE	—	NE	—	NE
PTSD	NE	—	NE	—	NE	—	NE
Others (PD, gender dysphoria)	NE	—	NE	—	NE	—	NE

*ADD/ADHD* attention deficit disorder/attention-deficit/hyperactivity disorder, *aOR* adjusted odds ratio, *ASD* autism spectrum disorder, *CG* comparison group, *CI* confidence interval, *NDD-UNS* neurodevelopmental disorder, unspecified, *NE* not reliably estimable, *OB* obesity, *OCD* obsessive-compulsive disorder, *OW* overweight, *PD* personality disorders,* PTSD*  post-traumatic stress disorder, *UW* underweight

For anxiety, adolescents with OW and OB had higher odds than those in the CG (aOR = 1.83, 95% CI: 1.24–2.71 and aOR = 2.10, 95% CI: 1.30–3.39, respectively). For depression, the association observed for OW in the unadjusted analysis was attenuated after adjustment and was no longer statistically significant (aOR = 1.45, 95% CI: 0.98–2.17), whereas adolescents with OB continued to have higher odds of reporting depression (aOR = 2.31, 95% CI: 1.47–3.63). UW estimates for anxiety and depression could not be obtained because no cases were observed in this group.

For eating disorder, adolescents with OW had lower odds than those in the CG (aOR = 0.30, 95% CI: 0.09–0.95), but the overall association between BMI category and eating disorder was not statistically significant. Adjusted associations for ASD, bipolar disorder, NDD-UNS, OCD, PTSD, and other psychiatric diagnoses were not reliably estimable because of the small number of events and/or separation in the multivariable models. These diagnoses were therefore retained primarily as descriptive findings in the unadjusted analyses.

## Discussion

This study identified several major findings regarding the associations between BMI and adolescent health outcomes, which are summarized as follows. Approximately 88% of participants fell within the WHO-defined normal BMI range. Among the extreme BMI categories (UW, OW, and OB), OW and OB were most common, while UW—particularly among adolescent girls—was underrepresented. After adjustment for age, gender, and country of residence, several associations between BMI category and physical and mental health outcomes remained. Adolescents with OW and OB had higher odds of reporting asthma, while OB was additionally associated with allergies and higher pain intensity. OW was associated with higher odds of thyroid disease. Regarding mental health, adolescents with OW and OB had higher odds of reporting anxiety, whereas OB was also associated with higher odds of depression and ADD/ADHD. Several associations observed in the unadjusted analyses were attenuated after adjustment. Findings involving the small UW group and rare outcomes should be interpreted cautiously because of sparse data and limited precision.

The WHO defines normal BMI for-age for children and adolescents aged 5–19 years as ranging from − 2 to + 1 standard deviations (SD) from the WHO Growth Reference median [59]. Thinness is defined as < − 2 SD, overweight as > + 1 SD, and obesity as > + 2 SD. Given the rapid biological changes of adolescence, this range is designed to accommodate natural BMI variability while identifying potential health risks without over-medicalization. However, “normal” BMI can differ markedly between individuals due to genetic, geographic, cultural, dietary, and lifestyle factors [60–63]. In adults, a well-established “J”-shaped relationship between BMI and health risks has been described [17, 23, 64–66], and this pattern may also be relevant during adolescence, when weight-related health problems may influence long-term physical and mental health trajectories [8, 23, 67, 68]. Notably, many health risks observed during this period remain modifiable, highlighting adolescence as a critical window for early detection and prevention.

The BMI distribution observed in this multinational sample is consistent with previous findings from high-, middle-, and low-income countries [69–72]. The prevalence of OB in our sample (3.7%) was somewhat lower than some other global estimates from 2016 [8] (5.6% in adolescent girls and 7.8% in boys), potentially reflecting regional differences or selection bias. UW prevalence in our study was low (0.34% overall; 0.56% in adolescent boys and 0.20% in girls) and substantially below global averages, which may also be influenced by non-response bias or cultural variability in body composition norms.

The association between higher BMI and increased pain perception supports prior literature linking OB to musculoskeletal stress, joint overload, and back pain in youth [73–79]. Importantly, the association with pain intensity persisted after adjustment for age, gender, and country of residence, with adolescents with OB reporting higher adjusted mean pain scores than those in the CG. Adolescents with OB also had higher odds of reporting pain “all the time” rather than “never” in the adjusted analysis. Nevertheless, the overall effect size for the unadjusted difference in pain intensity was negligible, indicating that the magnitude of the observed difference should not be overstated. In addition, previous research has linked obesity to abdominal pain, neuropathic pain, and chronic inflammatory states [49, 51, 80–82]. These findings support the hypothesis that both mechanical and immunological factors contribute to elevated pain burden in adolescents with higher BMI.

Somatic health complaints varied across BMI groups. The association between OB, OW and asthma is consistent with previous studies linking excess body weight to asthma risk [83–86]. This association persisted after adjustment for age, gender, and country of residence, with adolescents with both OW and OB showing higher odds of reporting asthma than those in the CG. This association may involve both mechanical factors (e.g., reduced lung volume, airway narrowing) [82–86] and inflammatory mechanisms mediated by adipokines such as leptin [81, 86–89]. Adolescents with OB also had higher adjusted odds of reporting allergies. This finding is consistent with previous research suggesting a possible relationship between childhood obesity and allergic or atopic conditions, potentially involving obesity-related low-grade systemic inflammation and alterations in immune regulation [90]. However, evidence for allergic conditions other than asthma remains less consistent, with associations varying according to the specific allergic phenotype examined. Therefore, given that allergies were self-reported as a broad category in the present study, this finding should be interpreted cautiously and does not allow conclusions regarding specific allergic diseases.

Cancer/tumour diagnosis and epilepsy were more frequently reported among adolescents with UW in the unadjusted analyses. However, both findings were based on only two cases among 16 underweight adolescents and should therefore be considered descriptive rather than evidence of robust associations. Moreover, the cross-sectional design does not allow the temporal direction of these relationships to be determined.

Some gastrointestinal differences were observed in the unadjusted analyses, including more frequent diarrhea among adolescents with OW and constipation among those with OB. For diarrhea, the higher odds among adolescents with OW persisted after adjustment, but the overall adjusted association with BMI category was not statistically significant; for constipation, no adjusted association was observed. Therefore, the present findings provide limited evidence for an independent association between BMI category and these gastrointestinal symptoms. Previous research has linked excess weight to functional gastrointestinal disorders [91–94], possibly due to altered gut microbiota, low dietary fiber intake, sedentary behavior, bile acid dysregulation, or low-grade inflammation [95–107].

The higher prevalence of thyroid disorders among adolescents with OW persisted after adjustment for age, gender, and country of residence, with adolescents with OW showing higher odds of thyroid disease than those in the CG. This finding is consistent with earlier findings of altered thyroid function in this population [108–112]. Mechanisms may involve hypothalamic–pituitary axis dysregulation, leptin-mediated hormone alterations, or inflammation-related changes in iodide transport and thyroid hormone synthesis [109–111, 113–119].Finally, the findings indicate associations between higher BMI and several psychiatric outcomes. After adjustment for age, gender, and country of residence, adolescents with OW and OB had higher odds of reporting anxiety than those in the CG. The association between OW and depression observed in the unadjusted analysis was attenuated after adjustment and was no longer statistically significant, whereas adolescents with OB continued to have higher odds of reporting depression. Higher rates of anxiety and depression among adolescents with OW and OB reported in previous studies may reflect interactions between biological vulnerability, psychosocial stress, sleep disruption, lifestyle factors, and weight stigma [24, 25, 120–130]. In some cultural contexts, emphasis on academic success over emotional health may further exacerbate distress [20]. Adolescents with OB also had higher adjusted odds of reporting ADD/ADHD than those in the CG. This finding aligns with existing evidence on shared neurobiological and behavioral pathways, including impulsivity, sleep disturbances, genetic overlap, and inflammation [131–138]. Although research on underweight status in ADHD is more limited, some studies have noted possible associations [135, 139]. However, the estimate for UW in the present study was imprecise because of the small number of underweight participants.

### Practical implications

The findings of this study carry important implications for both clinical practice and public health policy. The observed associations between BMI categories and a range of somatic and psychiatric conditions underscore the need for routine BMI monitoring during adolescence, not merely as a measure of weight status but as a potential indicator of broader health risk. Clinicians should consider BMI alongside physical health, mental well-being, nutritional status, and lifestyle factors as part of a comprehensive assessment of adolescent health.

From a policy standpoint, the results support the implementation of integrated prevention and early intervention strategies that address the dual burden of undernutrition and OB. Effective approaches should combine nutritional education, promotion of physical activity, early mental health screening, and equitable access to healthcare services, tailored to local infrastructure, resources, and sociocultural contexts. From a global public health perspective, the need for such integrated strategies is especially pronounced across countries with differing levels of economic development and healthcare infrastructure, where variations in healthcare access, diagnostic practices, and stigma related to obesity and mental health may further exacerbate health disparities. For instance, the higher-income countries in this study (United States, Sweden) generally offer more comprehensive access to pediatric mental health services and specialized nutritional care, whereas in the middle-income countries included (Serbia, Morocco, Vietnam), resource constraints and differing diagnostic infrastructures may result in under-recognition of both psychiatric comorbidities and the somatic conditions examined in this study. Similarly, stigma surrounding obesity and mental illness may manifest differently across these sociocultural contexts, potentially affecting both help-seeking behavior and the willingness to disclose such conditions, even in anonymous self-report surveys. As no country-level analyses were performed in this study, these represent plausible contextual considerations for interpreting the findings rather than empirically established country-specific patterns, and warrant dedicated investigation in future cross-national research.

These findings highlight the value of a holistic, cross-sectoral approach to adolescent health, integrating weight status with broader physical and mental health considerations.

### Strengths and limitations

This study has several limitations that warrant consideration. First, the MeSHe project employs a cross-sectional design, which precludes causal inference. Although associations were identified between BMI categories and various health conditions, the temporal direction of these relationships remains unclear. BMI status may act as a risk factor, a consequence of underlying disorders or treatments, or reflect shared biological, behavioral, or environmental mechanisms. Although the adjusted analyses accounted for age, gender, and country of residence, residual confounding by unmeasured factors and reverse causation cannot be excluded, highlighting the need for longitudinal studies to clarify these relationships.

Second, the use of online self-report questionnaires, while logistically efficient and scalable, introduces several potential sources of bias. Responses may be influenced by recall bias or social desirability, affecting the accuracy of self-reported weight, height, and health conditions [140]. Self-reported height and weight are known to be subject to reporting bias in adolescents, with prior studies indicating a tendency to underreport weight and overreport height, particularly among girls and adolescents with higher BMI [141]. This may have led to some degree of BMI misclassification in our sample; however, as no measured anthropometric data were available, the direction and extent of any such bias cannot be determined empirically. It should also be noted that the survey instrument was designed to capture broader mental and somatic health during the COVID-19 pandemic rather than to serve as a validated BMI assessment tool. Additionally, online data collection may have introduced some degree of selection bias, as participation required internet access and basic digital literacy. While this was unlikely to pose a significant barrier in most participating countries, it may have been more relevant in certain contexts, potentially leading to the underrepresentation of adolescents from more socioeconomically disadvantaged backgrounds. Moreover, those who chose to participate may differ systematically from non-respondents in overall motivation or engagement with health-related research, which could limit the broader generalizability of the findings. Furthermore, because both psychiatric diagnoses and somatic conditions were self-reported, via the same instrument, shared method variance may have inflated some associations independent of any true clinical relationship; For example, if respondents with a general tendency toward heightened symptom endorsement were more likely to report both psychiatric and somatic items. This form of common-method bias is distinct from the anthropometric reporting bias discussed above, as it concerns correlation among self-reported outcomes rather than misclassification of BMI exposure; as no independent or objective measures were available, it cannot be formally excluded.

An important limitation concerns the very small UW group, which included only 16 adolescents. This substantially reduced statistical power and precision for comparisons involving UW and resulted in sparse or zero-event cells for several outcomes. Consequently, some adjusted estimates could not be reliably obtained because of sparse data or separation in the regression models. This was particularly relevant for rare somatic and psychiatric outcomes. Findings involving UW and other rare outcomes should therefore be interpreted cautiously and, where reliable adjusted estimates could not be obtained, primarily as descriptive observations.

Gender distribution also presents a potential source of bias. A greater proportion of adolescent girls completed the survey, consistent with previous findings showing that girls are generally more likely to participate in survey-based research during adolescence [142–144]. This imbalance may have influenced prevalence estimates and associations, particularly for outcomes known to vary by gender. However, gender was included as a covariate in the adjusted analyses. Girls and boys may also differ in how they perceive and disclose symptoms, which may have contributed to gender-specific patterns in reported prevalence, independent of the method-variance effects described above. Additionally, adolescents who identified as non-binary were excluded due to their small number and the absence of appropriate BMI-for-age reference standards aligned with non-binary identities. Since gender identity does not map directly onto existing sex-based WHO reference categories, we were unable to reliably classify these participants, which limits the inclusivity and comprehensiveness of our findings.

A key strength of this study is its multinational design, which included adolescents from both high- and middle-income countries, representing a variety of cultural, economic, and healthcare system contexts. This broad geographic scope enhances the external validity of the findings and supports a more comprehensive understanding of how BMI categories relate to adolescent health across diverse settings. By including participants from countries with differing levels of infrastructure and public health resources, the study offers preliminary insights into the broader, system-level factors that may shape the relationship between BMI and health outcomes. At the same time, this cross-national approach introduces important limitations. Although country of residence was included as a covariate in the adjusted analyses, no country-specific analyses were conducted, and contextual factors—such as cultural norms related to body image, dietary patterns, healthcare access, or diagnostic practices—were not systematically accounted for in the analyses. These unmeasured differences may have influenced both BMI distribution and the reporting or recognition of health conditions, contributing to heterogeneity in the observed associations. Consequently, the findings should be interpreted with caution and not overgeneralized to specific national or regional contexts.

Finally, this study adopted a comprehensive, biopsychosocial approach to adolescent health by examining pain, somatic conditions, and mental health outcomes within the same multinational sample. In addition, the use of multivariable analyses adjusting for age, gender, and country of residence allowed the associations between BMI and health outcomes to be examined beyond unadjusted group comparisons. This holistic perspective is essential for guiding the development of sustainable interventions that are responsive to the complex and interconnected needs of adolescents across the BMI spectrum.

## Conclusions

This study examined associations between BMI categories and self-reported pain, somatic diseases, and psychiatric disorders in a multinational adolescent sample. The findings suggest that higher BMI categories were associated with distinct patterns of physical and mental health outcomes. OB was associated with higher pain intensity and persistent pain, while OW and/or OB were linked to a higher prevalence of self-reported somatic and psychiatric conditions.

These results underscore the importance of considering BMI within the broader physical and mental health assessment of adolescents rather than as an isolated indicator of weight status. They support the need for early identification and coordinated, preventive strategies that integrate nutritional, psychological, and lifestyle factors to address the needs of this vulnerable population.

## Data Availability

The datasets generated and/or analyzed during the current study are available from the corresponding author on reasonable request.

## Abbreviations

ADD: Attention-deficit disorder
ADHD: Attention-deficit/hyperactivity disorder
ASD: Autism spectrum disorder
BMI: Body mass index
CG: Comparison group
M: Mean
MeSHe: Mental and Somatic Health without borders
OB: Obesity
OW: Overweight
PTSD: Post-traumatic stress disorder
SD: Standard deviation
TSH: Thyroid-stimulating hormone
UW: Underweight
WHO: World Health Organization
